# Optimal Screening for Hereditary Head and Neck Paraganglioma in Asymptomatic SDHx Variant Carriers in the Netherlands

**DOI:** 10.1055/s-0044-1781438

**Published:** 2024-03-01

**Authors:** Anouk Frederique Heesters, Carli Tops, Thomas Potjer, Eleonora P.M. Corssmit, Jean-Pierre Bayley, Erik Hensen, Jeroen Jansen

**Affiliations:** 1 Department of Otorhinolaryngology, Leiden University Medical Centre, Leiden, The Netherlands; 2 Department of Clinical Genetics, Leiden University Medical Centre, Leiden, The Netherlands; 3 Department of Endocrinology, Leiden University Medical Centre, Leiden, The Netherlands; 4 Department of Human Genetics, Leiden University Medical Centre, Leiden, The Netherlands

**Keywords:** paraganglioma, hereditary, SDHA, SDHB, SDHD, screening

## Abstract

**Background** SDHx variant carriers have an increased risk of developing head and neck paraganglioma. The Dutch guidelines state that these patients require lifelong follow-up, but no clear recommendation is made about the frequency of screening.

**Objective** To determine the annual risk of developing head and neck paraganglioma in SDHx variant carriers after a negative initial screening.

**Methods** We conducted a retrospective single-center cohort study in the Netherlands that included 49 SDHA, SDHB, and SDHD variant carriers with a negative first screening and at least one follow-up. The main outcome measure was the annual risk of developing a paraganglioma for the SDHx variants separately.

**Results** Between 2000 and 2022, nine patients developed a paraganglioma all of whom were carriers of a SDHD variant ( *n* = 23). Neither the 24 SDHB-related cases nor the 2 SDHA variant carriers developed a paraganglioma after a median of 4.83 and 5.92 years of follow-up, respectively.

**Conclusion** The 5-year risk for head and neck paragangliomas in pathological SDHx variant carriers is less than 20%. A 5-year interval for screening SDHx carriers seems sufficient to prevent the unnoticed development of head and neck paragangliomas that warrant treatment.

## Introduction

Head and neck paragangliomas are rare, slow-growing, and usually benign neuroendocrine tumors related to the parasympathetic nervous system. The most common type is the carotid body tumor located in the carotid bifurcation, followed by the vagal tumors arising along the vagal nerve, and the jugulotympanic tumor arising from the jugular bulb or on the middle ear promotory. 1 2 Since head and neck paragangliomas rarely produce catecholamines (<4%), most symptoms are caused by tumor mass. 3 4 The most frequently observed sign is an indolent mass that in the ear is accompanied by pulsatile tinnitus and hearing loss. Occasionally, cranial nerve palsy (VII–XII) is the presenting sign of a paraganglioma. 5

In the Netherlands, 90% of the patients with head and neck paraganglioma have a pathogenic variant in the SDH gene encoding for the SDHA, SDHB, SDHC, SDHD, or SDHAF2 subunits. 6 SDHD and SDHAF2 have a parent-specific penetrance; paragangliomas are only present after paternal transmission. 7 The most common pathogenic variants are found in SDHD and SDHB. SDHD variant carriers have the highest risk of developing a paraganglioma, almost invariably developing a paraganglioma during their lifetime. 8 9 The lifetime penetrance of paragangliomas in SDHB variant carriers is approximately 40%, whereas the SDHA variant shows the lowest penetrance of all major PGL predisposition genes: approximately 10%. 10

The Dutch guideline for head and neck paraganglioma recommends radiological follow-up with a frequency of 2 to 5 years in case of a proven paraganglioma. In asymptomatic patients with a pathogenic variant in one of the SDH genes, a lifelong screening is recommended as well, but without recommendation about the frequency of screening. 11 Thus, there is no clear approach for screening for head and neck paraganglioma in asymptomatic SDHx variant carriers. Our policy in the Leiden University Medical Centre (LUMC) is to screen these mutation carriers every 2 to 3 years. If the first screenings are negative, a longer interval will be applied.

We previously showed that the prevalence of occult paragangliomas in asymptomatic carriers of SDHD is high (60%), compared with SDHB gene variants (12%). 12 However, this study was limited to the first radiological screening of the patient group and did not explore the risk of developing paragangliomas after the initial screening. A similar study in France from 2021 screened a large cohort of asymptomatic SDHx pathogenic variant carriers and did include more than one follow-up. 13 A total of 124 patients had at least one additional screening and 8% of them developed head and neck paraganglioma or sympathetic paraganglioma during a median period of 5 (1–13) years. Tufton et al reviewed all published recommendations regarding the follow-up and noted that there is little guidance on the frequency. 14 They concluded that the differences in phenotype and penetrance should be taken into account during follow-up and suggested a whole-body magnetic resonance imaging (MRI) only for carriers of the SDHB pathogenic variant with a 3-year interval. Finally, in the recently published international consensus statement a recommendation for all SDHx pathogenic variant carriers was made. This included a head and neck MRI every 2 to 3 years for all SDHx pathogenic variants, based on the authority of the participating experts. 15

To fill the knowledge gap that prevents the data-driven development of guidelines, our current study aims to determine the risk of developing head and neck paraganglioma in SDHx pathogenic variant carriers after a negative initial screening.

## Materials and Methods

### Study Design

We performed a retrospective single-center cohort study at the LUMC, where we retrospectively collected a dataset of patients with a SDHx pathogenic variant and a negative initial radiological screening for head and neck paraganglioma.

### Ethical Approval

The Medical Research Involving Human Subjects Act (Dutch abbreviation: WMO) does not apply to the current mentioned study. Therefore, it was exempt from review by the Medical Ethics Review Committee. The non-WMO Review Committee declared to have no objections to the study.

### Data Collection

The database of the Laboratory for Diagnostic Genome Analysis of the LUMC was used to identify SDHx carriers from 2000 to 2022. Only patients without a tumor during their initial workup and at least one radiological follow-up were included in this study. Asymptomatic SDHD variant carriers through maternal transmission were excluded. Subsequently, information about the remaining patients and their screening outcomes were extracted from the patient files. All data were collected in Castor (Castor EDC).

### Data Analysis

All data were analyzed using SPSS Statistics (IBM). Qualitative variables were expressed as numbers and percentages and the quantitative variables as median and range. We used the Kruskal–Wallis and chi-square tests to compare the different variant groups. Kaplan–Meier curves were used to present the annual risks regarding developing paragangliomas and symptoms. To compare the risk between the different types of variants, a log-rank test was performed. Hazard ratios were calculated through a Cox regression model to identify possible risk factors and confounders, such as age and gender.

## Results

### Population

**Fig. 1:**
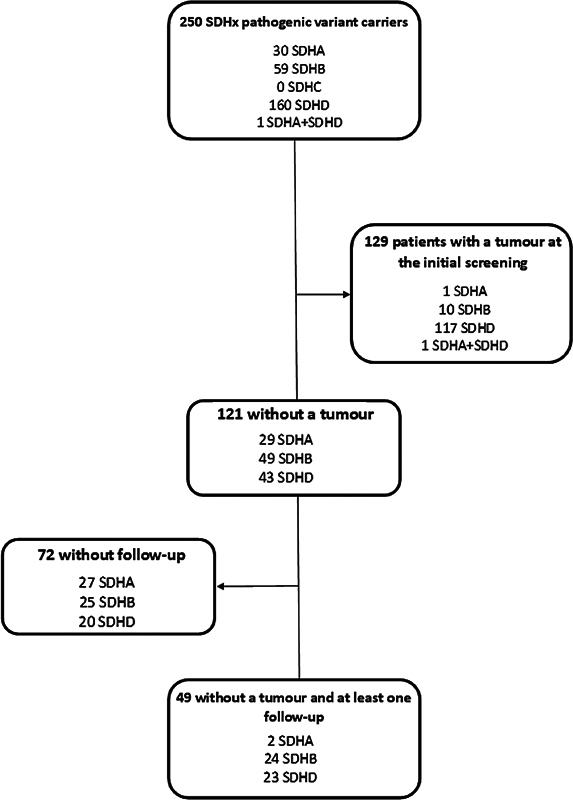
Inclusion flow chart.

As seen in Fig. 1 , a total of 49 subjects with a negative initial screening for paragangliomas and at least one follow-up were identified at the LUMC; 2 SDHA, 24 SDHB, and 23 paternally inherited SDHD variant carriers. All cases had a family member with paraganglioma. Two of the SDHB variants where classified as having an unknown significance.

### Subject Characteristics

The characteristics of the asymptomatic SDHx variant carriers are shown in Table 1 . The SDHB group was first screened at the median age of 43 years compared with 30 years in the SDHD group and 25 years in the SDHA group. However, this difference did not reach statistical significance.

**Table 1:** Characteristics of asymptomatic SDHx mutation carriers

	SDHA ( *N* = 2)	SDHB ( *N* = 24)	SDHD ( *N* = 23)
Gender (%)			
Female	2 (100)	19 (79.2)	11 (45.8)
Age at initial negative screening (median, range)	25.00 y (16–34)	43.00 y (12–75)	30.00 y (16–64)
Tumor at MRI (%)	0 (0)	0 (0)	9 (39.1)
Age at discovery tumor (median, range)	–	–	39.00 y (24–69)
Tumors (%)			13
Carotid	–	–	8 (61.5)
Vagal	–	–	4 (30.8)
Jugular	–	–	1 (7.7)
Symptoms (%)	0 (0)	0 (0)	1 (11.0)
Death	0 (0)	0 (0)	0 (0)
Follow-up (median, range)	4.09 y (3.00–5.17)	5.92 y (2.33–13.33)	4.83 y (1.08–17.17)

### Clinical Outcomes

As presented in Table 1 , nine subjects, exclusively from the SDHD group, developed at least one tumor at a median age of 39 years (24–69). The median time interval between the negative initial screening and the discovery of a tumor was 6.08 years with a minimum and maximum of 4.17 and 14.08 years, respectively. In these patients, the median time between their initial and second screening was 4.83 years (0.92–7.75), whereas the overall median time interval between the initial and second screening was 4.58 years (0.92–17.17). Most of the tumors were carotid body tumors (61.5%), followed by vagal (30.8%) and jugulotympanic paraganglioma (7.7%). Only one of the patients developed symptoms related to their tumor, 3.25 years after the discovery of the paraganglioma. The overall median follow-up period of the subjects without a tumor was 5.54 years (1.08–17.7); 4.09 years (3.00–5.17) for SDHA, 5.92 years (2.33–13.33) for SDHB, and 4.83 years (1.08–17.17) for SDHD variant carriers.

### Annual Risk

**Fig. 2:**
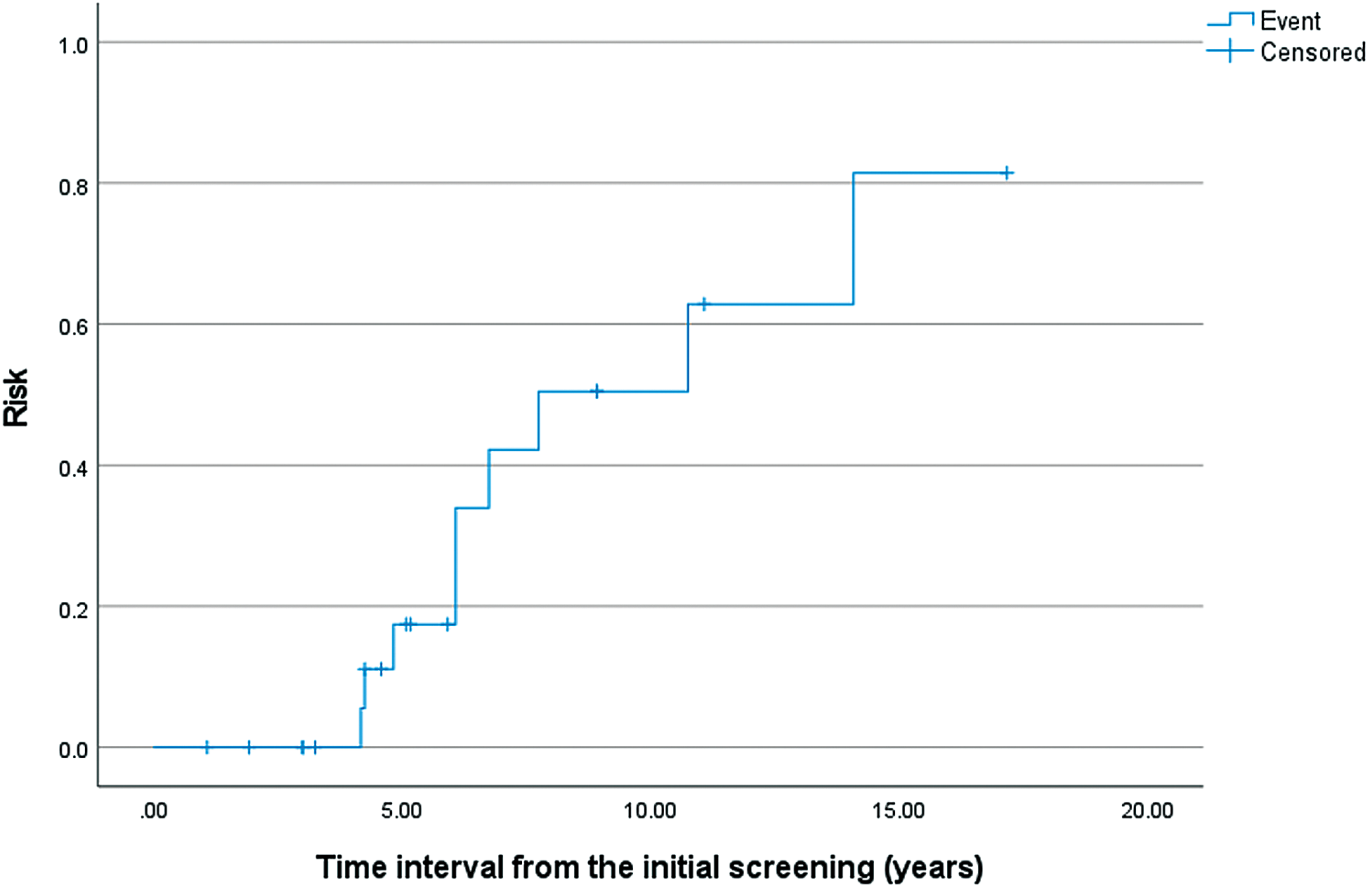
Inverted Kaplan–Meier curve presenting the annual risk of developing a paraganglioma for SDHD variant carriers (family cases).

Among the SDHD variant carriers, the risk of developing a head and neck paraganglioma was 17.5% (confidence interval [CI]: 15.9–19.1) after 5 years, 62.9% (CI: 33.5–92.3) after 10 years, and 82.4% (CI: 52.8–112.0) after 15 years ( Fig. 2 ).

## Discussion

Asymptomatic carriers of pathogenic SDHx variants are advised lifelong screening for paragangliomas. As expected, our study showed a significantly higher risk of developing head and neck paragangliomas in SDHD variant carriers compared with other SDHx genes. In fact, no paragangliomas occurred at all during the follow-up of the SDHA and SDHB group. Of the total study population, 18% developed a paraganglioma, which is higher than the prevalence in the similar study from France (8%). 13 This difference in prevalence is probably due to the high proportion of SDHD variant carriers in the Netherlands.

In the SDHD group, the calculated 5-year risk for developing a paraganglioma was 17.5% and the first paraganglioma was discovered 4.17 years after their initial screening. In addition, the development of symptoms after radiological diagnosis will typically last several years, if any symptoms occur at all, as shown in our results. Hence, we consider MRI screening with an interval of 5 years sufficient for this group. Since the SDHB group did not develop paraganglioma during a median follow-up time of 5.92 years, the screening interval could be longer. However, to prevent loss of follow-up it may be advisable to have a similar 5-year screening interval. Although there were only two patients in the SDHA group, it is unlikely that the risk exceeds that of SDHB given the fact that the overall penetrance of SDHA is estimated to be only 10%, compared with 42% in SDHB. 10 16

In our study, the patients developed a tumor at the maximum age of 69 years, which accords with a larger study at the LUMC from 2011, presenting a maximum risk of 87% at the age of 70 in SDHD pathogenic variant carriers. 17 This could argue for reducing the screening frequency or even discontinuing the screening in older patients. In addition, a negative correlation between age and the development of new symptoms has already been demonstrated. 18 Therefore, in older patients, the benefits of early detection could no longer outweigh the burdens of screening or treatment. Future studies determining the risk of developing paraganglioma specifically in elderly patients are needed to assess these assumptions.

The major limitation of this study is the small sample size. Only patients who were screened and treated in the LUMC were included in this study. By including more medical centers, a larger study population could be provided. This will increase the power of the study and strengthen our conclusions.

## Conclusion

Given the fact that paraganglioma syndromes have an age-dependent, incomplete penetrance and that the tumors have slow growth rates, we propose that frequent radiological follow-up of the head and neck region is an unnecessary burden to the healthy carriers of an SDHx pathogenic variant. MRI screening every 5 years seems to be a safe interval for these patients.
